# The Golgin Family of Coiled-Coil Tethering Proteins

**DOI:** 10.3389/fcell.2015.00086

**Published:** 2016-01-11

**Authors:** Tomasz M. Witkos, Martin Lowe

**Affiliations:** Faculty of Life Sciences, University of ManchesterManchester, UK

**Keywords:** golgin, Golgi apparatus, tether, vesicle trafficking, cytoskeleton

## Abstract

The golgins are a family of predominantly coiled-coil proteins that are localized to the Golgi apparatus. Golgins are present in all eukaryotes, suggesting an evolutionary conserved function. Golgins are anchored to the Golgi membrane by their carboxy terminus and are predicted to adopt an extended conformation that projects into the surrounding cytoplasm. This arrangement is ideal for the capture or tethering of nearby membranes or cytoskeletal elements. Golgin-mediated tethering is thought to be important for vesicular traffic at the Golgi apparatus, the maintenance of Golgi architecture, as well as the positioning of the Golgi apparatus within cells. In addition to acting as tethers, some golgins can also sequester various factors at the Golgi membrane, allowing for the spatiotemporal regulation of downstream cellular functions. Although it is now established that golgins are membrane and cytoskeleton tethers, the mechanisms underlying tethering remain poorly defined. Moreover, the importance of golgin-mediated tethering in a physiological context remains to be fully explored. This review will describe our current understanding of golgin function, highlighting recent progress that has been made, and goes on to discuss outstanding questions and potential avenues for future research with regard to this family of conserved Golgi-associated proteins.

## Introduction

The Golgi apparatus is the central organelle of the secretory pathway of eukaryotic cells. It is comprised of flattened membrane discs called cisternae that are typically layered on top of one another to form the characteristic Golgi stack (Klumperman, 2011; Lowe, 2011). In most vertebrate cells the Golgi stacks are laterally connected to form the Golgi ribbon, which is flanked on either side by tubular *cis*- and *trans*-Golgi networks, which serve as the entry and exit stations of the Golgi, respectively (Klumperman, 2011; Lowe, 2011). The Golgi apparatus is a major site of post-translational modification. Resident enzymes present within the Golgi cisternae act sequentially upon secretory cargo to add and/or process glycan chains on glycoproteins and glycolipids as they transit the Golgi stack from one side to the other (Stanley, 2011). The Golgi also acts as a major sorting compartment, packaging cargo for delivery to various downstream destinations as well as returning selected proteins back to the endoplasmic reticulum, the first station of the secretory pathway, via retrograde trafficking (Spang, 2011; Guo et al., 2014).

Several mechanisms have been proposed for cargo trafficking within the Golgi stack, with the cisternal maturation model currently favored (Glick and Luini, 2011). In this model, secretory cargo is retained within the Golgi cisternae, which migrate and progressively mature as they transit the Golgi stack. Maturation occurs via retrograde transport of resident proteins including the glycan processing enzymes, which are retrieved from later cisternae via COPI-mediated vesicular retrograde traffic. Vesicle and tubule-based anterograde transport mechanisms have also been proposed, which may be relevant for trafficking of certain cargoes (Glick and Luini, 2011). Interestingly, a recent study showed that COPI, in addition to driving retrograde vesicle formation, may also function in tubule-based anterograde traffic (Park et al., 2015).

The vertebrate Golgi ribbon is typically positioned adjacent to the centrosome, the major microtubule-organizing center (Yadav and Linstedt, 2011). This localization is mediated by interactions with microtubules, the minus end-directed microtubule motor dynein, and physical attachment to the centrosome itself. In contrast, plants and fungi have discrete Golgi stacks whose positioning is dependent upon the actin cytoskeleton as opposed to microtubules (Faso et al., 2009). In those species examined, the Golgi apparatus appears to be surrounded by a “matrix” that excludes the majority of other cytoplasmic components (Lowe, 2011; Xiang and Wang, 2011). The matrix is believed to comprise numerous proteins that associate with the cytoplasmic face of Golgi membranes and extend into the cytoplasm to form a dense mesh-like structure. The matrix is thought to confer structural integrity to the Golgi and is likely important for other Golgi functions including vesicular trafficking.

The golgins are thought to be major constituents of the Golgi matrix. Golgins are proteins that are predominantly coiled-coil in nature that are attached via their extreme carboxy-terminus to the cytoplasmic face of the Golgi apparatus (Munro, 2011). These features allow the golgins to extend over a significant distance (predicted to be between 100 and 600 nm, depending on the particular golgin) into the cytoplasm, an ideal characteristic for catching or tethering other membranes such as transport vesicles or Golgi cisternae, or cytoskeletal elements. Interestingly, the coiled-coil regions are interrupted by numerous short breaks, which may allow a high degree of flexibility in golgin conformation (Ishida et al., 2015).

**Figure 1:**
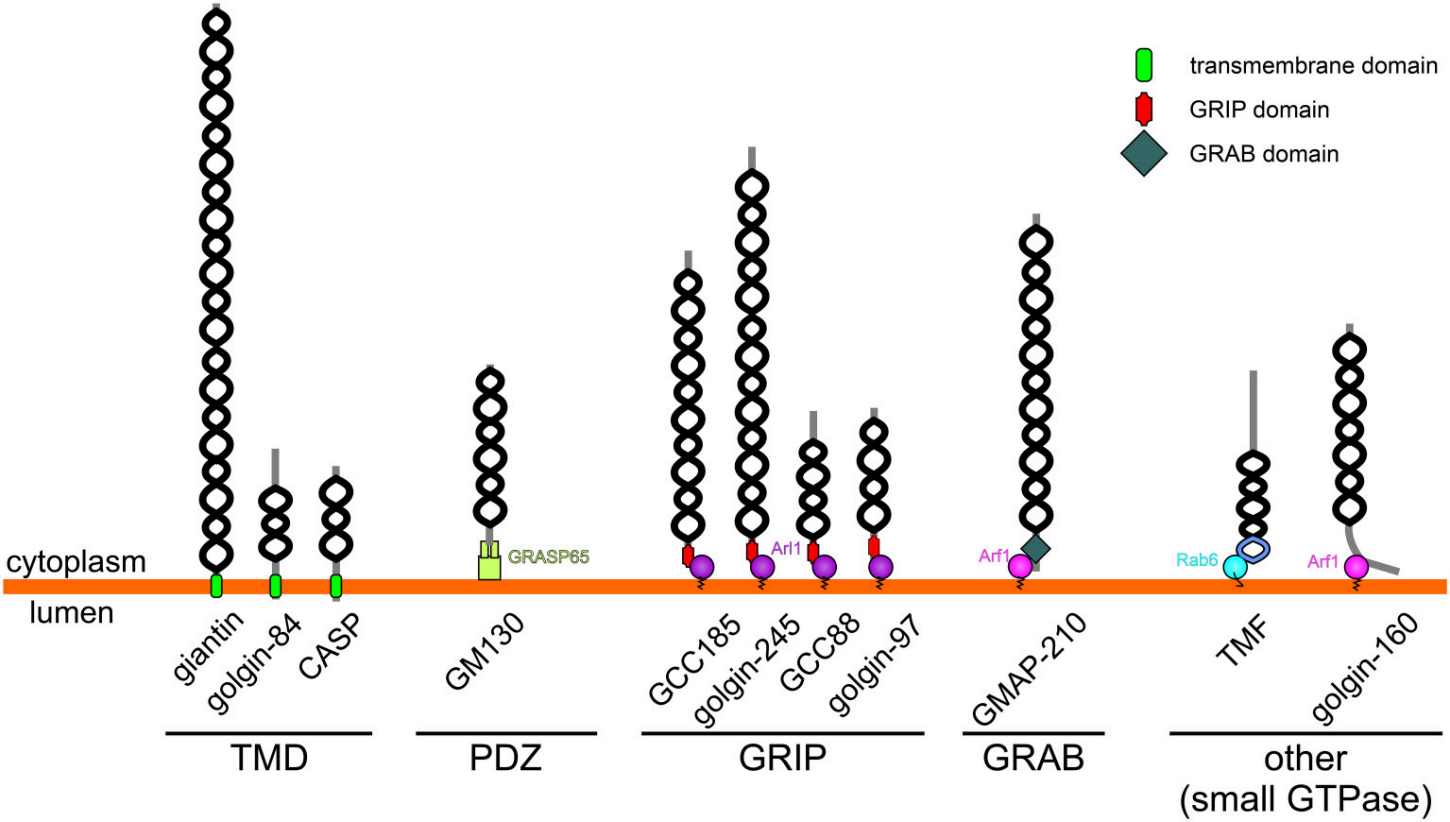
**Topology and membrane attachment of golgins**. Golgins are comprised predominantly of coiled-coil regions (depicted by black helices; note that the frequent short breaks found within the coiled-coil regions of golgins are not shown for simplicity). Golgins can either be directly anchored to the Golgi membrane through carboxy-terminal transmembrane domains (giantin, golgin-84 and CASP), or they can associate with the Golgi membrane through interactions with other Golgi-localized proteins. The carboxy-terminus of GM130 acts as a ligand for the PDZ domains of GRASP65. *Trans*-Golgi golgins have a GRIP domain that interacts with Arl1, whereas GMAP-210 has an analogous GRAB domain that binds to Arf1. TMF and golgin-160 are attached to the Golgi membrane via binding of their carboxy-terminal regions to Rab6 or Arf1 respectively.

There are at least 11 canonical golgins that conform to the description above (Munro, 2011; Table 1 and Figure 1; note that Golgi proteins that contain globular domains or large regions of non-coiled-coil, such as p115, are not defined as golgins according to this description). Although the golgins share the same general features, they lack significant sequence homology with one another. They also localize to distinct regions of the Golgi, consistent with their ability to mediate distinct tethering events. As may be expected given their important role in maintaining Golgi organization and membrane traffic, golgins have been conserved throughout the eukaryotic kingdom. The “higher” eukaryotes, such as vertebrates, have the highest complement of golgins, reflecting the more varied cell types and trafficking events that occur in those species, but they are also present in other metazoans and in single cell organisms such as yeast (Table 1).

**Table 1:** **The golgin family of tethering proteins**.

**Golgin**	**Homologs in**					**Golgi** **localisation**	**Confirmed tethering of**		**Anchor for^a^**
	**Fish**	**Fly**	**Worm**	**Plant**	**Yeast**		**vesicles**	**cytoskeleton**	
**GMAP210**	✓	✓	✓	✓	✓		✓		
									Cdc42
									Tuba
**GM130**	✓	✓	✓	✕	✓	*cis* Golgi	✓	✓	RasGRF
									Stk25
									GABARAP
									WAC
**Golgin-160**	✓	✕	✕	✕	✕			✓	
**Giantin**	✓	✓	✕	✕	✕			✓	ACBD3
**CASP**	✓	✕	✓	✓	✓	Golgi rims			
**Golgin-84**	✓	✓	✓	✓	✕		✓		
**TMF**	✓	✓	✓	✓	✓		✓		
**GCC88**	✓	✓	✓				✓		
**GCC185**	✓	✓	✓	✓^b^	✓	*trans* Golgi		✓	
**Golgin-97**	✓	✓	✕				✓		
**Golgin-245**	✓	✓	✓				✓		

a *Anchor indicates binding of the indicated cytoplasmic factor to the golgin, which is typically important for the regulation of a downstream cellular process. Factors for vesicle or cytoskeleton tethering are not shown in this column*.

b *Plant AtGRIP shows the highest similarity to the GRIP domains of GCC185 and golgin-97*.

In addition to sharing the same general architecture, most golgins are also able to associate with small GTPases of the Rab, Arf, or Arl families (Munro, 2011). These GTPases are enriched in distinct Golgi regions and contribute to membrane identity by recruiting various effector proteins to these regions. Therefore, in some cases small GTPase binding mediates both the membrane attachment and targeting of the golgin to a distinct Golgi region (see Table 1 and Figure 1). For example GRIP domain golgins are targeted to the *trans*-Golgi through association with Arl1, which is enriched at this location. In other cases, such as for the tail-anchored golgins (golgin-84, giantin, CASP), the significance of small GTPase binding remains to be determined, although it is likely to play a role in membrane tethering as discussed further below. How the tail-anchored golgins are recruited to distinct Golgi regions remains to be determined, but is likely to depend upon the properties of the trans-membrane domain and flanking cytoplasmic region, and possible association with binding partners (Misumi et al., 2001; Gillingham et al., 2002).

## Golgins as membrane tethers

The elongated nature of golgins lends itself to the prediction that they function as tethers, reaching out into the cytoplasm to capture transport vesicles and possibly other membranes. Tethering could be followed by membrane fusion to complete vesicular transport or generation of cisternae, or not, in the case of stable cross-linking of membranes as occurs during stacking of Golgi cisternae. Despite this attractive idea, direct experimental evidence for golgins acting as tethers has been relatively scarce until recently. The first indication that golgins can indeed tether vesicles came from *in vitro* analysis using purified golgins and isolated vesicle fractions (Malsam et al., 2005). Tethers comprising golgin-84 and CASP, or GM130 and p115, were shown to capture COPI vesicles, with each tether (golgin-84-CASP or GM130-p115) capturing a different population of vesicles (Malsam et al., 2005). In addition to showing that tethers can capture vesicles, this study also provided the first evidence that golgins can contribute to specificity of vesicle trafficking. This view has been cemented by a recent landmark study that systematically studied the ability of different golgins to tether transport vesicles in intact cells (Wong and Munro, 2014). Using an elegant gain-of-function assay, golgins were shown to be sufficient for the tethering of transport vesicles *in vivo*, with different golgins tethering distinct classes of transport vesicle (Wong and Munro, 2014). For example, *trans*-Golgi localized golgins were able to tether incoming endosomally-derived transport vesicles, but not those mediating ER to Golgi traffic, while the converse was true for some of the *cis*-Golgi golgins. Thus, golgins contribute to the specificity of vesicle-mediated trafficking. Interestingly, a few golgins appeared unable to mediate tethering in this assay (giantin, CASP, GCC185), suggesting that they may not function as vesicle tethers, that they mediate tethering of vesicle classes that were not in the analysis, or perhaps that they mediate tethering of non-vesicle membranes e.g., cisternae.

**Figure 2:**
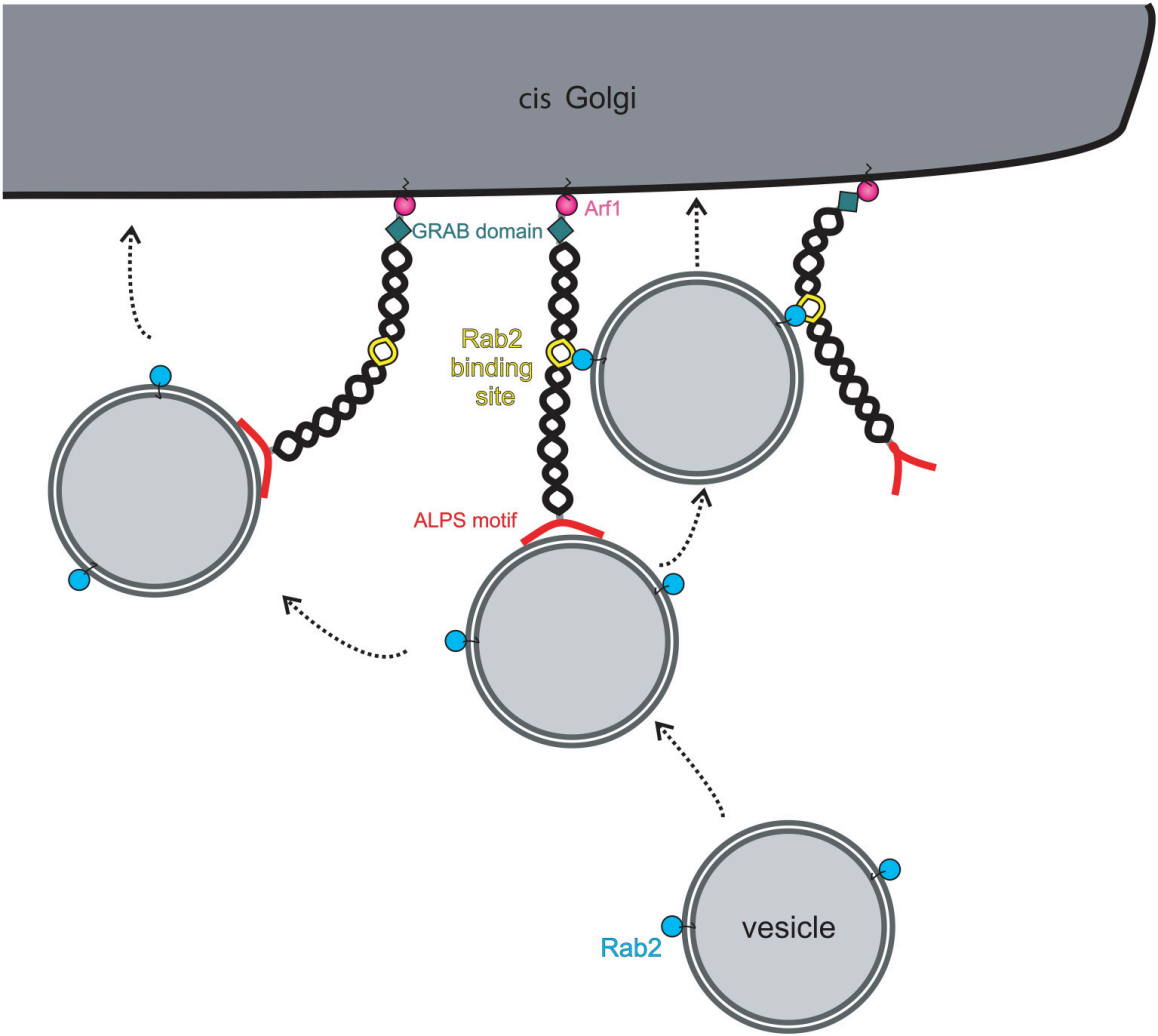
**Model for vesicle tethering by the golgin GMAP-210**. Vesicles are specifically recognized and captured at the *cis*-Golgi by the amino-terminal ALPS motif (red). Bound vesicles can be transferred to a neighboring golgin(s) through additional vesicle attachment sites present on the neighboring golgin(s). Alternatively, the presence of other vesicle attachment sites on the same golgin will allow transport of vesicles along the same golgin molecule. In the case of GMAP-210, the vesicle attachment site is a Rab2-binding site, allowing binding to vesicle-associated Rab2. Movement onto the secondary vesicle attachment sites will bring the vesicle into closer proximity with the Golgi membrane to promote fusion there. GMAP-210 may also undergo a conformational change to bring the vesicle into closer proximity to the Golgi membrane, as shown on the left.

Although the evidence that golgins can function as vesicle tethers is compelling, in nearly all cases the underlying mechanisms remain to be elucidated. The best-characterized golgin in terms of its ability to tether vesicles is GMAP-210, which has an amphipathic lipid packing sensor (ALPS) motif at its extreme amino terminus (Figure 2). The ALPS motif is able to sense membrane curvature, and in the case of GMAP-210 is able to mediate attachment to lipid vesicles which have a high degree of inherent curvature (Drin et al., 2007). Indeed, using purified liposomes, Antonny and colleagues were able to show the ability of GMAP-210 to asymmetrically tether high curvature liposomes to those of low curvature, mimicking the tethering of transport vesicles to Golgi cisternae (Drin et al., 2008). Not only did this provide strong support that GMAP-210 is a membrane tether, it also revealed a potential mechanism for tethering. Subsequent studies showed that indeed the GMAP-210 ALPS motif is able to tether transport vesicles in cells (Pranke et al., 2013; Sato et al., 2015), and moreover, despite only binding to membrane lipids, that it can impart selectivity to the tethering process (Pranke et al., 2013).

The tethering mechanism used by other golgins, which lack an ALPS motif, is less well defined. GM130 is able to associate with the vesicle docking protein p115 via its extreme amino-terminus (Nakamura et al., 1997). This interaction has been proposed to mediate tethering of ER-to-Golgi transport vesicles and tethering of Golgi vesicles during post-mitotic Golgi assembly, but direct evidence for this is currently lacking. Although GM130 lacking the p115 binding site is deficient in post-mitotic Golgi assembly (Nakamura et al., 1997), it was still able to tether ER-derived transport vesicles in the *in vivo* tethering assay described above (Wong and Munro, 2014). Further study will be required to address this apparent discrepancy. For other golgins, the tethering mechanisms remain obscure. It has been proposed that binding to Rab GTPases is involved, since all golgins are able to associate with Rabs (Sinka et al., 2008). Rab binding sites tend to be located along the length of golgins (Diao et al., 2003; Sinka et al., 2008; Hayes et al., 2009), rather than being localized at the ends, as might be expected for optimum tethering efficiency. Rab binding may therefore act secondary to the initial tethering event, as has been shown for GMAP-210 (Sato et al., 2015; (Figure 2). However, for intra-Golgi transport, where vesicles bud close to where they are to be tethered on adjacent cisternae, binding at the membrane-distal end of the golgin may not be required. Attachment to membrane-proximal Rab binding sites may be sufficient to tether the vesicle close to the site of budding, holding it near the fusion site on the adjacent cisterna and preventing diffusion out of the Golgi matrix. Tethering at the membrane-distal ends may be utilized more for capture of vesicles delivered from other organelles. In this case, sequential binding to the Rab binding sites along the length of golgins would promote the passage of a tethered vesicle through the Golgi matrix, funneling it toward the correct membrane domain where it will ultimately fuse (Munro, 2011). The sequential and specific transfer of a vesicle from one golgin to another, mediated by golgin-Rab interactions, would therefore help ensure the fidelity of vesicle delivery at the Golgi apparatus.

Some golgins are able to associate with the multi-subunit tethering complex called COG, suggesting a degree of cooperation between the two types of Golgi vesicle tethers (Sohda et al., 2010; Miller et al., 2013). How the two tethers function together is currently unclear, but one may envisage a sequential mode of action, or alternatively a mechanism where the two tethers act in parallel to ensure a higher degree of efficiency and specificity in vesicle tethering (Willett et al., 2013). Interactions between golgins and SNARE proteins, which mediate membrane fusion downstream of tethering, have also been reported. GM130 and its binding partner p115 bind to *cis*-Golgi SNAREs (Shorter et al., 2002; Diao et al., 2008) while GCC185 can bind to syntaxin 16 involved in trafficking to the *trans*-Golgi (Ganley et al., 2008). Again, how tethering is coupled with fusion is poorly understood, and whether other golgins also physically associate with SNAREs to couple tethering and fusion remains to be determined.

It has been proposed that golgins act in a largely redundant manner to tether transport vesicles (Munro, 2011). This view is supported by the ability of different golgins to tether vesicles containing the same cargo, and by the mild or sometimes absent effects of depleting or knocking out golgin function in cultured cells or in model organisms such as yeast or flies. There is evidence for redundancy in golgin function in ER-to-Golgi traffic (Roboti et al., 2015), and it is likely to occur for other Golgi trafficking steps. However, complete redundancy in golgin function is unlikely to occur in all cells or for all cargo proteins. Although redundancy between golgins complicates their analysis, it is important to remember that such redundancy is likely to be physiologically meaningful. For example, knockout of GMAP-210 in mice leads to a lethal skeletal dysplasia, which mimics the achondrogenesis type IA disease that occurs in humans with loss of function mutations in GMAP-210 (Smits et al., 2010). Interestingly, Golgi organization and traffic is affected only in certain cell types, explaining the tissue-selective nature of the disease, most likely due to compensation or redundancy of GMAP-210 in the unaffected cells. Similarly, mice lacking functional giantin display a chondrodysplasia (Katayama et al., 2011), while knockout mice lacking TMF1 are more susceptible to infection of their gut due to defects in secretory mucin production (Bel et al., 2012, 2014). These studies indicate that to fully appreciate the importance of golgin-mediated tethering, it will be important to use a variety of experimental approaches and model systems, and to analyze a wide spectrum of cargoes in a range of different cell types.

## Golgins as cytoskeleton tethers

The Golgi apparatus is intimately associated with the cytoskeleton, with the microtubule network playing a key role in both the organization and positioning of the Golgi in vertebrate cells (Yadav and Linstedt, 2011). A number of proteins are thought to link the Golgi to the microtubule cytoskeleton, amongst which are members of the golgin family. In this sense the golgins can be thought of as microtubule tethers. GMAP-210 was initially shown to link the Golgi to microtubules (Infante et al., 1999), and subsequently to the centrosome (Rios et al., 2004), but these findings are contentious, with more recent studies suggesting that GMAP-210 acts exclusively as a membrane tether (Gillingham et al., 2004; Drin et al., 2008; Wong and Munro, 2014; Sato et al., 2015). Other golgins do, however, appear to function in Golgi-microtubule linking. GM130 and GCC185 promote microtubule nucleation at the *cis*- and *trans*-Golgi respectively. GM130 recruits the nucleation-promoting factor AKAP450 to the *cis*-Golgi to promote microtubule growth there (Rivero et al., 2009), while GCC185 recruits CLASP2, a microtubule plus-end binding protein that stabilizes new microtubule growth, to the *trans*-Golgi (Efimov et al., 2007). In both cases, the Golgi-nucleated microtubules are important for maintenance of Golgi ribbon organization and the directed secretion that occurs during polarized cell migration (Efimov et al., 2007; Rivero et al., 2009).

More recently, GM130 was shown to contribute to microtubule dynamics during mitosis (Wei and Seemann, 2009), when the vertebrate Golgi undergoes extensive fragmentation to facilitate its equal partitioning into the two daughter cells (Wei and Seemann, 2009). In early mitosis, GM130 sequesters importin-α, leading to activation of the microtubule spindle assembly factor TPX2 at Golgi membranes, which in turn causes nucleation of spindle microtubules at the Golgi (Wei et al., 2015). Additionally, GM130 can bind directly to microtubules to further stabilize Golgi-microtubule attachment (Wei et al., 2015). These events are thought to ensure the equal segregation of Golgi membranes via attachment to the mitotic spindle, in a manner reminiscent to that used for chromosomes.

In addition to binding to microtubules, golgins can also associate with the microtubule motor protein dynein. In Drosophila, the golgin lava lamp, whose closest vertebrate homolog is giantin, binds to dynein and the microtubule associated factors p150 glued and CLIP-190 (Papoulas et al., 2005). These interactions are required for dynein-based motility of Golgi membranes that is required for cellularization during embryogenesis. It has been known for many years that the vertebrate Golgi requires dynein for its pericentrosomal localization and ribbon integrity, but the nature of the Golgi membrane-dynein association has remained elusive. However, a recent study showed that golgin-160 is major determinant for Golgi recruitment of dynein (Yadav et al., 2012). Golgin-160 is anchored to the Golgi membrane via a carboxy-terminal interaction with the Arf1 GTPase, while the distal amino-terminus binds to dynein, linking the motor directly to the Golgi membrane. The interaction between golgin-160 and dynein is functionally important, as indicated by impaired Golgi motility, positioning and ribbon integrity upon its disruption.

## Golgins as protein anchors

Because golgins are elongated proteins that extend into the cytoplasm, they can sequester proteins from the surrounding cytoplasm. Indeed, GM130 is able to bind and sequester a number of cytoplasmic proteins including the Cdc42 guanine nucleotide exchange factor (GEF) Tuba, and Ras guanine nucleotide releasing factor (GRF; Kodani et al., 2009; Baschieri et al., 2014). Tuba is proposed to promote localized Cdc42 activation at the Golgi (Kodani et al., 2009). Although RasGRF is a negative regulator of Cdc42 activation, its interaction with GM130 is thought to sequester the protein to prevent it from inhibiting Cdc42 at Golgi membranes, again leading to a localized activation of the Golgi pool of Cdc42 (Baschieri et al., 2014). This localized activation of Cdc42 is thought to be important for polarized cell migration. Interestingly, RasGRF can activate Ras and downstream MAP kinase signaling, suggesting that an additional function of the GM130-RasGRF interaction is to limit mitogenic signaling. Indeed loss of GM130 has been reported to promote cell growth, which may be relevant during tumourigenesis (Baschieri et al., 2014).

In addition to promoting localized Cdc42 activation at the Golgi apparatus, GM130 can also contribute to cell migration by scaffolding another protein, the protein kinase Stk25, also known as YSK1 (Preisinger et al., 2004). GM130 recruits Stk25 to Golgi membranes and is required for its localized activation there. Stk25 in turn is important for Golgi positioning and directed cell migration, and is especially important for polarized membrane delivery in neurons. Loss of GM130 or Stk25 leads to a failure to correctly polarize the Golgi in neurons with reduced membrane delivery and impaired dendrite specification and growth (Matsuki et al., 2010).

A recent study has revealed yet another example of GM130 recruiting cytoplasmic factors to the Golgi. In this case, GM130 binds to the autophagy inducer GABARAP and its negative regulator WAC at the Golgi apparatus (Joachim et al., 2015). The dual sequestration of these factors is thought to be important for the regulation of autophagy, with GM130 acting as a negative regulator of this process.

Giantin binds to the cell fate determinant ACBD3 (Acyl-CoA Binding Domain containing 3), also known as GCP60 (Sohda et al., 2001). ACBD3 acts in conjunction with Numb to specify cell fate during asymmetric cell division, as occurs during neurogenesis. ACBD3 is sequestered at the Golgi by giantin in interphase, but when cells divide it is released into the cytoplasm, whereupon it can be partitioned asymmetrically to drive neuron specification in one of the daughter cells (Zhou et al., 2007). How ACBD3 is released from giantin in mitosis is currently unclear.

## Regulation of golgin function

**Figure 3:**
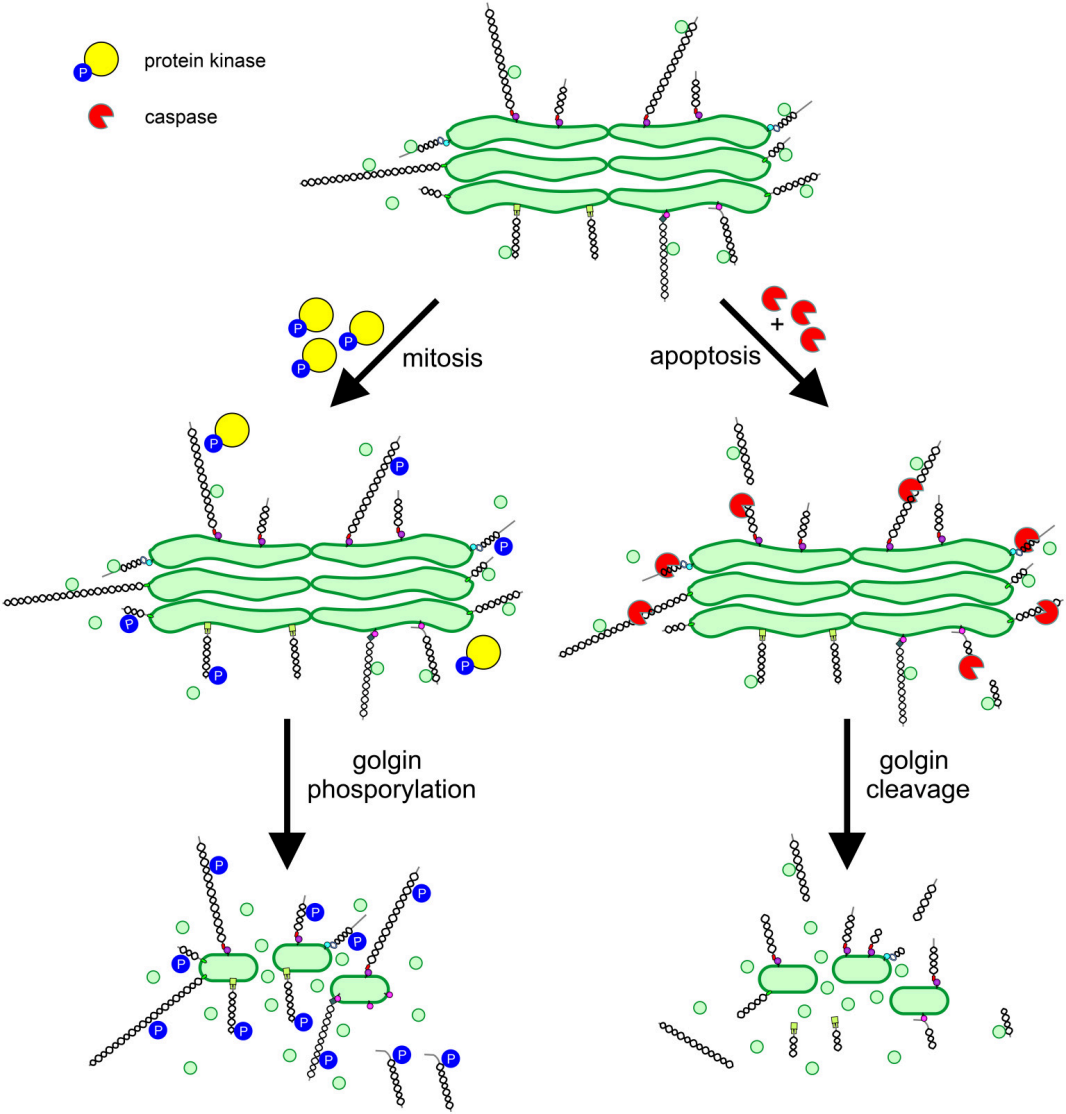
**Regulation of golgin function in mitosis and apoptosis**. Golgins primarily act as vesicle tethers at the Golgi apparatus. In mitosis, golgins undergo phosphorylation mediated by protein kinases, which impairs vesicle tethering by preventing interaction with binding partners e.g., p115 binding to GM130, or by disassociation of the golgin from the Golgi membrane e.g., golgin-160. In apoptosis, activated caspases cleave golgins leading to their release from the Golgi e.g., GM130, or their extensive trimming e.g., golgin-160. Phosphorylation or cleavage of golgins prevents vesicle tethering, leading to conversion of cisternal membrane to vesicles and Golgi apparatus disassembly.

As described above the vertebrate Golgi apparatus undergoes extensive fragmentation in mitosis, converting to clusters of vesicles and tubules dispersed in the cytoplasm (Wei and Seemann, 2009). The dramatic reorganization of the Golgi that occurs in mitosis is coincident with an arrest in secretory trafficking. Both processes are driven by protein phosphorylation, with key structural and trafficking components acting as targets for mitotic kinases. The Golgi stacking proteins GRASP55 and GRASP65 undergo hyperphosphorylation in mitosis, promoting cisternal unstacking (Barr et al., 1997; Jesch et al., 2001). Strikingly, the majority of the other (non-GRASP) Golgi phosphoproteins are golgins. All golgins studied to date undergo mitotic phosphorylation (Nakamura et al., 1997; Diao et al., 2003, 2008; Yadav et al., 2012), which likely plays a major role in promoting Golgi disassembly. It was proposed several years ago that Golgi vesiculation arises from continued vesicle budding in the absence of tethering or fusion, converting cisternae to vesicles (Warren, 1985). The high degree of golgin phosphorylation is consistent with this model, if one assumes that phosphorylation inhibits the tethering activity of golgins (Figure 3). For GM130 such an effect has been demonstrated. Phosphorylation by the mitotic kinase CDK1 prevents binding to p115, preventing the tethering of p115-containing transport vesicles (Nakamura et al., 1997; Lowe et al., 1998). Whether the phosphorylation of other golgins inhibits tethering remains to be demonstrated. Interestingly, phosphorylation of golgin-160 in mitosis triggers its release, together with dynein, from the Golgi membrane (Yadav et al., 2012). The loss of Golgi-associated dynein in mitosis contributes to the dispersal of Golgi membranes and therefore Golgi partitioning in mitosis.

The vertebrate Golgi apparatus also undergoes extensive fragmentation in apoptosis, and again, fragmentation occurs alongside a block in secretory trafficking (Hicks and Machamer, 2005). However, differently to mitosis, Golgi fragmentation and transport arrest are caused by caspase-mediated proteolytic cleavage, which is an irreversible process. In line with the important role for golgins in maintaining Golgi structure and trafficking pathways, several golgins have been identified as caspase substrates (Hicks and Machamer, 2005). Caspase mediated cleavage of golgins would be expected to abolish tethering activity, which requires linking of one end of the golgin (bound to the vesicle) to the other (bound to the Golgi cisterna; Figure 3). However, this remains to be demonstrated experimentally.

## Conclusions and future directions

It has now been convincingly demonstrated that golgins are able to tether transport vesicles, and that by doing so they contribute to the specificity of membrane trafficking at the Golgi apparatus. The challenge now is to understand the mechanisms by which vesicle tethering occurs, and how tethering is linked to the downstream process of membrane fusion. It will also be important to understand how golgin-mediated tethering is coordinated with the activity of other tethers such as COG, and the downstream fusion machinery. Moreover, the range of different tethering events that each golgin can mediate remains to be determined. Open questions include: Are golgins capable of tethering more than one type of vesicle? Do golgins mediate tethering of cisternal membranes to one another for Golgi assembly? Can golgins participate in vesicle tethering at the same time as linking the Golgi to the cytoskeleton or anchoring cytosolic factors, or are these activities independent of one another? Further investigation will be required to address these issues.

Although there is redundancy between golgins, the degree to which this occurs is likely to vary between different golgins and cell types and for different secretory cargoes. To date, most analysis of golgin function has been carried out in tissue culture cells. To fully appreciate the physiological importance of golgins, it will be important to analyze them in animal models. A good example is that of GMAP-210, whose loss leads to achondrogenesis in humans and mice, attributable to specific impairment of secretory trafficking in chondrocytes (Smits et al., 2010). It would not have been possible to predict this result from cell-based work. Hence, for other golgins, animal studies are very likely to prove informative. Analysis of animal models should also provide new information about golgin function during development, as well as the extent to which redundancy exists between different golgins *in vivo*, which can be addressed using knockout animals lacking different combinations of golgins.

## Author contributions

ML wrote the article and helped design the figures. TW commented on the article, and designed and constructed the figures.

## Acknowledgments

TW is supported by a PhD studentship from the Wellcome Trust (096601/Z/11/Z) and a research grant from the MRC (MR/N000366/1). Work in the Lowe lab is supported by the Wellcome Trust, BBSRC, MRC and Lowe Syndrome Trust.

## Author note

During the editing of the proof for this review a paper was published showing that the flexible conformation of the golgin GCC185 is required for its function in vesicle tethering at the trans-Golgi. The same paper also showed that vesicle tethering is mediated by the extreme amino-terminus of GCC185 (Cheung et al., 2015).

### Conflict of interest statement

The authors declare that the research was conducted in the absence of any commercial or financial relationships that could be construed as a potential conflict of interest.
